# Monitoring modifiable injury risk factors over an in-season mesocycle in semi-professional female field hockey players

**DOI:** 10.1186/s13102-024-00814-8

**Published:** 2024-01-31

**Authors:** Violeta Sánchez-Migallón, Víctor Moreno-Pérez, Pablo Terrón-Manrique, Vicente Fernández-Ruiz, Catherine Blake, Archit Navandar, Álvaro López Samanes

**Affiliations:** 1 Faculty of Health Sciences, Universidad Francisco de Vitoria, Madrid, Spain; 2 Center for Translational Research in Physiotherapy, Department of Pathology and Surgery, Universidad Miguel Hernández, San Juan, Elche, Spain; 3 Institute for Sport and Health, University College Dublin, Dublin, Ireland; 4 School of Public Health, Physiotherapy and Sport Science, University College Dublin, Dublin, Ireland; 5 Faculty of Sports Science, Universidad Europea de Madrid, 28670 Villaviciosa de Odón, Madrid, Spain; 6 Faculty of Sport Sciences, Universidad Politécnica de Madrid, Madrid, Spain; 7 Education, Research Methods and Evaluation Department, Faculty of Human and Social Sciences, Universidad Pontificia Comillas, Madrid, Spain

**Keywords:** Team sports, Injury risk, Season, Neuromuscular, fatigue

## Abstract

**Objective:**

This study aimed to determine changes of modifiable injury risk factors and fatigue parameters during a mesocycle (4 months of the competitive season) in semi-professional female field hockey players (Spanish 2nd Division).

**Methods:**

Fourteen female field hockey players (age: 22.6 ± 4.9 years) participated in the study over 4 months of the competitive season (September–December 2019). The players were tested each month for their: maximal isometric knee flexion, hip adduction, and abduction muscle strength; passive straight leg raise and ankle dorsiflexion range of motion (ROM); countermovement jump height; and perceptual fatigue (through a perceived well-being questionnaire).

**Results:**

Statistical differences were reported in isometric knee flexion torque in the dominant and non-dominant limb (*p* = < 0.001, η_p_^2^ = 0.629,0.786 respectively), non-dominant isometric hip abductors torque (*p* = 0.016, η_p_^2^ = 0.266) and isometric hip adductors torque in dominant and non-dominant limbs (*p* = < 0.001, η_p_^2^ = 0.441–546). Also, significant differences were reported in the straight leg raise test (*p* = < 0.001, η_p_^2^ = 0–523, 0.556) and ankle dorsiflexion (*p* = 0.001, η_p_^2^ = 0.376, 0.377) for the dominant and non-dominant limb respectively. Finally, the jump height measured showed significant differences (*p* = <.001, η_p_^2^ = 0.490), while no differences were reported in perceived well-being parameters (*p* = 0.089–0.459).

**Conclusion:**

Increments in isometric muscle strength and fluctuations in ROM values and vertical jumping capacity are reported over an in-season mesocycle (i.e., 4 months of the competitive season)***.*** This information can be used to target recovery strategies to make them more efficient.

## Introduction

Field hockey is an intermittent team sport played in more than 125 countries worldwide, according to the International Field Hockey Federation, being included amongst the team sports disciplines that participate in the Olympic program [1]. This sport involves speed, agility and the ability to access a ground-level ball with a hockey stick and therefore places considerable effort on the lower limbs. Previous studies have reported that lower limb injuries account for 51% of all injuries in female field hockey players being thigh (18%) and hip/groin (12%) were regions identified as most susceptible to injury [2, 3] in the lower limbs. From a clinical viewpoint, the identification of meaningful risk factors is essential to implementing preventive strategies during female field hockey practice [4].

It is well recognized that the probability of suffering a muscle injury is determined by the interaction between several non-modifiable and modifiable extrinsic and intrinsic risk factors such as age [5], previous injury [3], reduced hamstring muscle strength [5], decreased knee extension range [6] decreased ankle dorsiflexion range of motion (ROM) [7] or the appearance of fatigue during training and matches [8].

Fatigue accumulation can result in a condition of overload and/or overtraining, which is associated with physiological consequences (e.g., reduction in muscle force production), as well as with an increased risk of injury [9]. Previous research has shown that acute fatigue appearance after one or consecutive matches has been related to changes in hip abductor and adductor maximal isometric muscle strength [10], neuromuscular control [11] and hip flexion ROM values [12] in field hockey players. However, the evidence for both the degree of variability and the value of controlling these variables over a competitive field-hockey season is scarce. Thus, it is paramount to provide empirical information to underpin the development of prevention strategies and correction programs by strength and conditioning coaches and physiotherapists.

Thus, this study aimed to evaluate changes of intrinsic modifiable field hockey injury risk factors such as ROM, isometric muscle strength, and neuromuscular performance measured by countermovement jump and physical/perceptual fatigue for 4 months during of competitive season in semi-professional female field hockey players.

## Material and methods

### Participants

Fourteen semi-professional female field hockey players took part in this study (age: 22.6 ± 4.9 years; weight: 63.4 ± 5.8 kg; height: 167.0 ± 0.1 cm; body mass index: 22.6 ± 1.7; field hockey training experience: 12.9 ± 5.8 years; training per week: 8.7 ± 1.1 hours included technical/tactical and physical training, divided in 3 days per week). All the female field hockey players played in a semi-professional team that competed in the 2nd Division of the Spanish Female Field Hockey League. Exclusion criteria were a) goalkeepers, b) female field hockey players who were not able to perform the test due to an injury, sickness, or any physical complaint were excluded from the final sample of this study. All players received information of the neuromuscular test battery procedure and signed a consent form for participating in this study. The experimental procedure of this study was in accordance with the guidelines outlined in the Declaration of Helsinki 2013 and was approved by the University Francisco de Vitoria Ethics Committee (number 45/2018).

### Experimental design

**Fig. 1:**
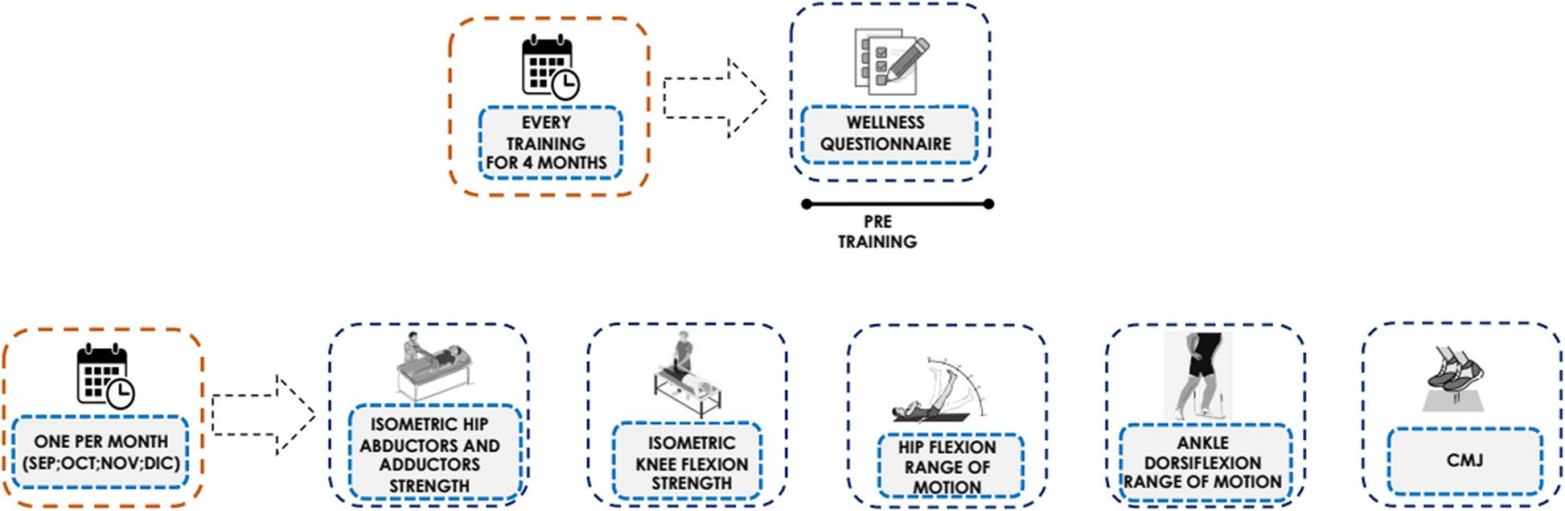
Experimental design

The current investigation is a cohort study, planned to be conducted monthly throughout the duration of the 2019–2020 season. However, the appearance of the COVID-19 pandemic in March 2020, caused the training and competition to be stopped immediately. Since the Spanish field-hockey season is paused during the winter months of January and February (an indoor competition is played during this time), data was recorded only from September 2019–December 2019. Thus, the assessments were performed at four different separate time points during the competitive season (Fig. 1): September 2019 (i.e., 3 weeks after the preseason period); October 2019 (i.e., after completing the first four matches of the season); November 2019 (i.e., after completing seven official competitive games); December 2019 (after completing the 11 official competitive games), a schedule which was predetermined based on player availability and match schedule. The players were tested before the training session, on the last day of the month in a physiotherapy room at the training facility of the field hockey club.

During each testing session, hockey players participated in anthropometric measurements, ROM tests (i.e., ankle dorsiflexion and straight leg raise test), and maximal isometric muscle strength tests (i.e., hip adduction and abduction and knee flexion) in their dominant and no dominant limbs). Also, fluctuations in their neuromuscular performance (i.e., measured by countermovement jump) were recorded. All testing were conducted by two senior sports physiotherapists (i.e., with 10 years and 6 years of experience respectively).

Perceptual fatigue was measured using a perceived well-being questionnaire recorded before each training session [13] to detect fatigue status and the mean for each session/week was recorded for subsequent analysis. In addition, each month female hockey players filled injury questionnaire and all the measurements were undertaken at the same time of the day (i.e., to avoid the influence of circadian rhythms) on neuromuscular performance that has been reported in other female team sports [14].

Before each testing session, female field hockey players conducted a standardized warm-up that consisted of 5 min of jogging, 5 min of dynamic stretching and joint mobility exercises [15]. All the female field hockey players were previously familiarized with the testing procedures because the muscle strength test employed in this investigation was part of an ongoing injury preventive program employed by the club which had been initiated a few months before this study. Based on the recommendations of Wollin et al. [15] the order in which the players were tested, and the selection of the tested leg were chosen randomly. Limb dominance preference was determined through a questionnaire. To minimize interference from uncontrolled variables, subjects were instructed to maintain their usual lifestyle and normal dietary intake on the days of measurement and to refrain from caffeine intake 24 h before the experiment [16].

#### Isometric knee flexion torque

Maximal isometric muscle knee flexion strength of hamstrings on both sides was measured using the previously described methodology [17] with a portable handheld dynamometer (Nicholas Manual Muscle Tester; Lafayette Indiana Instruments, Lafayette, IN, USA). For this measurement, each player was positioned in a prone position on a bench, with 15 degrees of knee flexion. The examiner (first physiotherapist) placed the dynamometer in the distal portion of the sural triceps using external belt fixation according to previous reported [18, 19], while the assistant (second physiotherapist) held the subject’s pelvis over the sacrum, to prevent elevation during the test. The examiner requested a knee flexion with the intention to bring the heel of the foot to the buttock. Female field hockey players performed voluntary contractions for a maximum of 5 seconds against the dynamometer and repeated the exercise twice for each leg. There was a rest period of 30 seconds between each measurement [18] and two repetitions were performed for both the dominant and non-dominant leg. The highest value obtained was recorded for subsequent analysis. The ICC for this test was 0.82 [19].

#### Isometric hip abductor and adductor torque

Maximal isometric strength of the hip abductors and adductors on both sides was measured using a portable handheld dynamometer (Nicholas Manual Muscle Tester; Lafayette Indiana Instruments, Lafayette, IN, USA). Female field hockey players were in a supine position with their hips in a neutral position and told to stabilize by holding onto the sides of the table. The first physiotherapist applied resistance in a fixed position, 5 cm to the proximal edge of the lateral for abduction or medial for adduction malleolus fixated with a rigid belt around the legs as previously reported [20], while the second physiotherapist maintain the correct position of the patient. Female field hockey players performed voluntary contractions for a maximum of 5 seconds against the dynamometer and repeated the exercise twice for each leg There was a rest period of 30 seconds between each measurement [21, 22]. Two repetitions were performed for both the dominant and non-dominant leg. The highest value obtained was recorded for subsequent analysis. The ICC for this test was 0.93–0.97 [20].

#### Hip flexion range-of-motion (straight leg raise test)

To measure the flexibility of the hamstrings through hip flexion ROM with the knee extended, the straight leg raise test was performed [18, 23]. An ISOMED Unilevel inclinometer (Portland, Oregon) with a telescopic extension long arm was used for the measurement. The inclinometer was placed approximately on the external malleolus and the distal arm was aligned parallel to an imaginary bisecting line of the limb [24]. The test ended with one or more of the following criteria: a) The examiner was unable to continue the joint movement evaluated, due to the high resistance developed by the stretched muscle group; b) The examinee reports a significant sense of distrust; c) noted compensations that could increase the measurement; d) the appearance of pain. The ICC for this test was 0.93–0.97 [25].

#### Ankle dorsiflexion range of motion (ROM)

Unilateral ankle dorsiflexion ROM was measured using the LegMotion System (LegMotion, Check your Motion, Albacete, Spain). Each female field hockey players took a standing position on the LegMotion System with their hands on the hips and the foot of the ankle being tested placed on the measurement platform. The contralateral foot was positioned off the platform with the toes positioned at the edge of the platform. While maintaining this position, players were instructed to flex the knee on the same leg as the tested ankle, with the knee contacting a metal stick placed in front of the toe. Once contact between the knee and stick, and between the heel and ground, were maintained for 3 s, the stick was progressively moved away from the knee in 1-cm increments each time until the knee could not contact the stick or heel contact with the ground could not be maintained [24] The furthest achieved distance (cm) of the metal stick from the closest toe was recorded. Two attempts were permitted for each ankle (dominant and non-dominant legs) with 10 s of passive recovery administered between attempts. The dominant leg for each player was determined as their preferred leg for kicking a ball [26, 27]. Additional attempts were performed until two ankle dorsiflexion measurements with less than 10% difference for each ankle were attained. The highest value of the two attempts for each ankle was used for further analysis. The ICC for this test was 0.96–0.98 [26].

#### Countermovement jump

Vertical jump height was measured following established procedures [28]. For measuring each jump a validated contact platform was used (Chronojump Boscosystem®, Barcelona, Spain) [29]. Specifically, female field hockey players performed a countermovement jump without arm swing with their hands on their hips. Each player performed two maximal attempts, with each attempt interspersed with 45 s of passive recovery. Additional attempts were performed until two jumps differed in height by less than 10% for each jump type. The greatest jump height (cm) for each jump type was used for analysis. The ICC for this test was 0.90 [30].

#### Perceived well-being questionnaire

Perceptual responses were measured using a psychological questionnaire developed by McLellan et al. (2010) designed to assess perceived well-being questionnaire (5-WQ). Field hockey players completed a 5-item psychometric questionnaire that included questions generally related to fatigue, perceived levels of sleep quality, muscle pain, stress level, and mood. A five-point Likert scale (i.e., values of 1–5 with 0.5 point increments) was used. Perceived well-being questionnaire was determined by summing the 5 questions to obtain a score ranging from 5 to 25 [13].

### Statistical analysis

Given the observational design and exploratory nature of the study involving volunteer participants, a priori sample size estimates for hypothesis testing were not carried out*.* However, where differences in the measured variables were observed over time, the magnitude of the effect size and *p*-value are reported.

Shapiro–Wilk test was first used to assess the normal distribution of data. The means and standard deviations of the data were determined. These were compared for the ROM, strength, countermovement jump and perceived well-being test variables registered in four different months (September, October, November, and December) using a repeated-measures analysis of variance (ANOVA). If significant differences were found between the measures across the four instances, a Bonferroni post hoc test was performed. In cases where the sphericity assumption was violated, a Greenhouse–Geisser adjustment for *p*-values was reported. The effect sizes of the repeated measures ANOVA were measured using partial η_p_^2^ values, and the following thresholds were used: trivial (η_p_^2^ ≤ 0.01), small (0.01 ≤ η_p_^2^ < 0.06), medium (0.06 ≤ η_p_^2^ < 0.14), and large (η_p_^2^ ≥ 0.14).

Perceived well-being values were compared using the non-parametric Friedman’s test of repeated measures, and effect sizes were determined using Kendall’s coefficient of concordance (Kendall’s W), the following thresholds were used: trivial (W ≤ 0.1), small (0.1 ≤ W < 0.3), moderate (0.3 ≤ W < 0.6) and large (W ≥ 0.6). Significance was set at *p* < 0.05. Statistical analysis was carried out using Jamovi (version 2.3.12, www.jamovi.org).

## Results

Differences were observed over time for each of the measured variables (Table 1), within this cohort of female hockey players. Testing protocol proved feasible and acceptable for the team roster and hockey players and we reported a very good data completeness due to the 14 female hockey players competed all testing days.

**Table 1:** The mean ± standard deviation for isometric strength, range-of-motion and countermovement jump variables measured over the 4 months of the mesocycle

Variables	September	October	November	December
Isometric knee flexion torque DOM (Nm)	238.76 ± 42.59*	267.41 ± 43.99*	257.1 ± 39.35*	331.92 ± 59.27
Isometric knee flexion torque NO-DOM (Nm)	230.05 ± 39.30*/***	292.97 ± 50.92*	254.71 ± 42.96*	329.15 ± 42.76
Isometric hip abductor strength torque DOM (Nm)	174.46 ± 31.92	188.69 ± 15.62	209.11 ± 32.00	213.14 ± 30.62
Isometric hip abductor strength torque NO-DOM (Nm)	175.84 ± 18.03	189.56 ± 23.36**	220.89 ± 30.98	215.14 ± 33.28
Isometric hip adductor strength torque DOM (Nm)	200.26 ± 34.88*	218.06 ± 47.85*/**	227.58 ± 50.72	243.89 ± 44.32
Isometric hip adductor strength torque NO-DOM (Nm)	200.23 ± 45.50*/**	208.32 ± 42.85*/**	233.12 ± 46.47	234.24 ± 49.32
Straight Leg Raise Test ROM DOM (°)	75.00 ± 8.89*	67.86 ± 8.17*	74.69 ± 8.56*	81.62 ± 11.72
Straight Leg Raise Test ROM NO-DOM (°)	75.36 ± 7.91*	70.00 ± 6.97*	75.31 ± 11.24*	84.10 ± 9.14
Ankle dorsiflexion ROM DOM (cm)	13.43 ± 3.16*	13.75 ± 3.00*	13.38 ± 3.10*	12.00 ± 3.18
Ankle dorsiflexion ROM NO-DOM (cm)	13.39 ± 3.16*	13.61 ± 2.99*	13.35 ± 3.11*	12.00 ± 3.03
CMJ (cm)	22.65 ± 3.39	24.49 ± 5.8	18.35 ± 4.27	24.28 ± 4.87

Abbreviations: *DOM* dominant, *NO-DOM* non-dominant, *ROM* range of motion, *°* degrees, *cm* centimetres, *CMJ* countermovement jump. * Statistical differences comparing December (*p* < 0.05). ** Statistical differences comparing November (*p* < 0.05).*** Statistical differences comparing October. (*p* < 0.05)

### Isometric knee flexion torque

**Fig. 2:**
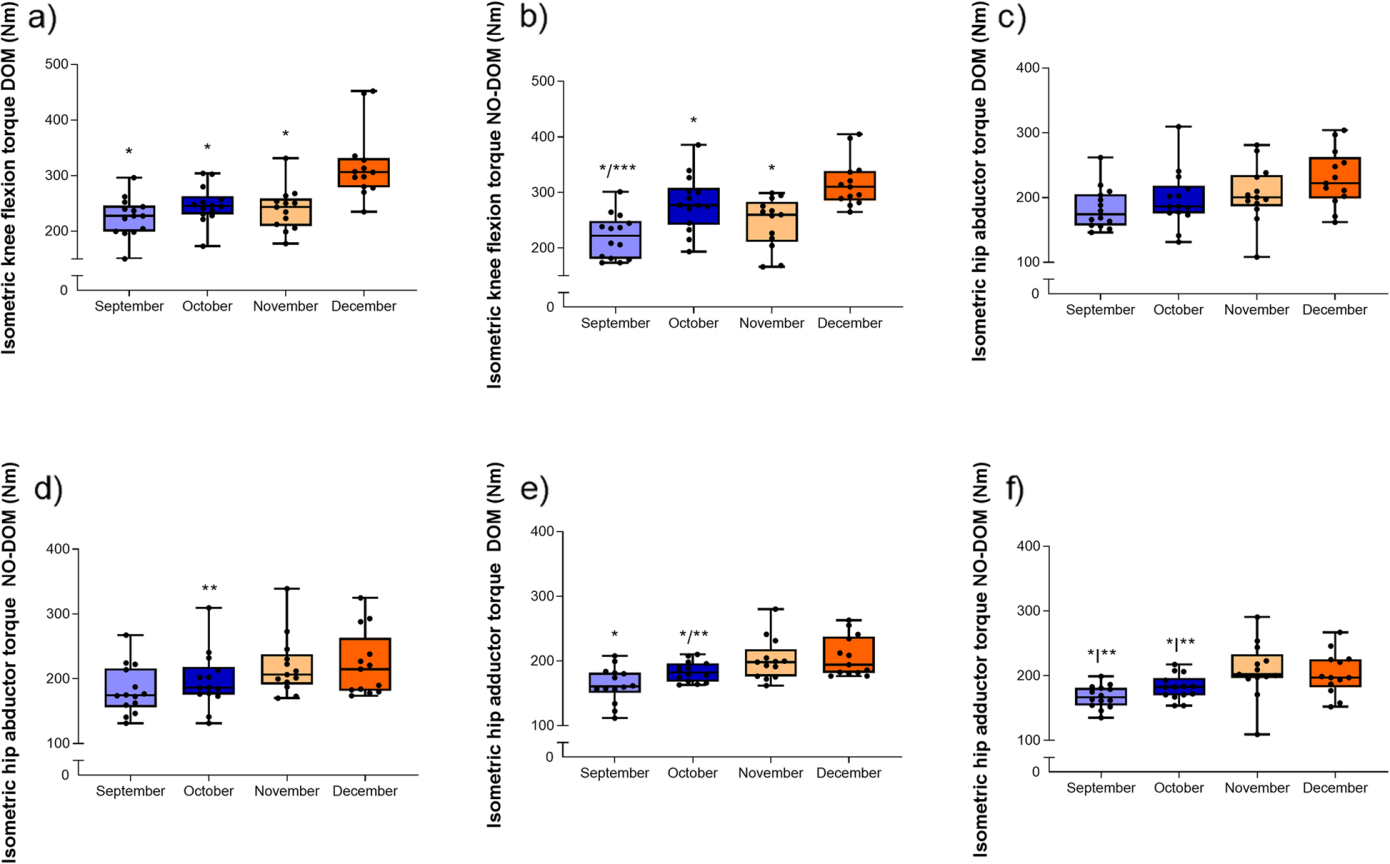
Isometric and hip abductor/adductor strength torque values in female field hockey players. Nm = newton/meter; DOM = Dominant; NO-DOM = non-dominant. * Significantly different when compared to December (*p* < 0.05). ** Significantly different when compared to November. (*p* < 0.05).*** Significantly different when compared to October. (*p* < 0.05)

Isometric knee flexion torque showed significant differences and a large effect size for the dominant limb (F(3,33) = 18.6, *p* = < 0.001, η_p_^2^ = 0.629), with the post hoc tests showing that month 4 (December) values were significantly higher than previous months (i.e., September, October and November) (*p* = 0.001–0.003) (Fig. 2a). Similarly, in the non-dominant limb, significant differences with a large effect size were also present (F_(3,33)_ = 40.3, *p* = <.001, η_p_^2^ = 0.786), with post-hoc analysis showing higher December values compared to previous months (i.e., September, October and November) (*p* = < 0.001–0.004) and also higher October values compared to September (*p* = < 0.001) (Fig. 2b).

### Isometric hip abductor and adductor torque

No significant changes with large effect sizes were seen in the isometric hip abductor muscle strength measurements for the dominant limb after post-hoc testing (F_(3,33)_ = 5.18, *p* = 0.051, η_p_^2^ = 0.320) (Fig. 2c) However, non-dominant limb showed a significant effect (F_(3,33)_ = 3.98, *p* = 0.016, η_p_^2^ = 0.266) when November values being significantly higher when compared to those from October (*p* = 0.032) (Fig. 2d).

Isometric hip adductors muscle strength showed significant differences and large effect size for the dominant limb (F_(3,33)_ = 8.67, *p* = < 0.001, η_p_^2^ = 0.441) (Fig. 2e), and post-hoc tests showed lower values at September values when compared to December (*p* < .001) and November (*p* = 0.028). Similarly, in the non-dominant limb, statistical differences were also reported (F_(3,33)_ = 13.2, *p* = < 0.001, η_p_^2^ = 0.546) (Fig. 2f). Values at December were higher than those at October (*p* = 0.035) and September (*p* = 0.024), and also higher at November compared to October (*p* = 0.002) and September (*p* = 0.012) respectively.

### ROM values (hamstring and ankle dorsiflexion)

**Fig. 3:**
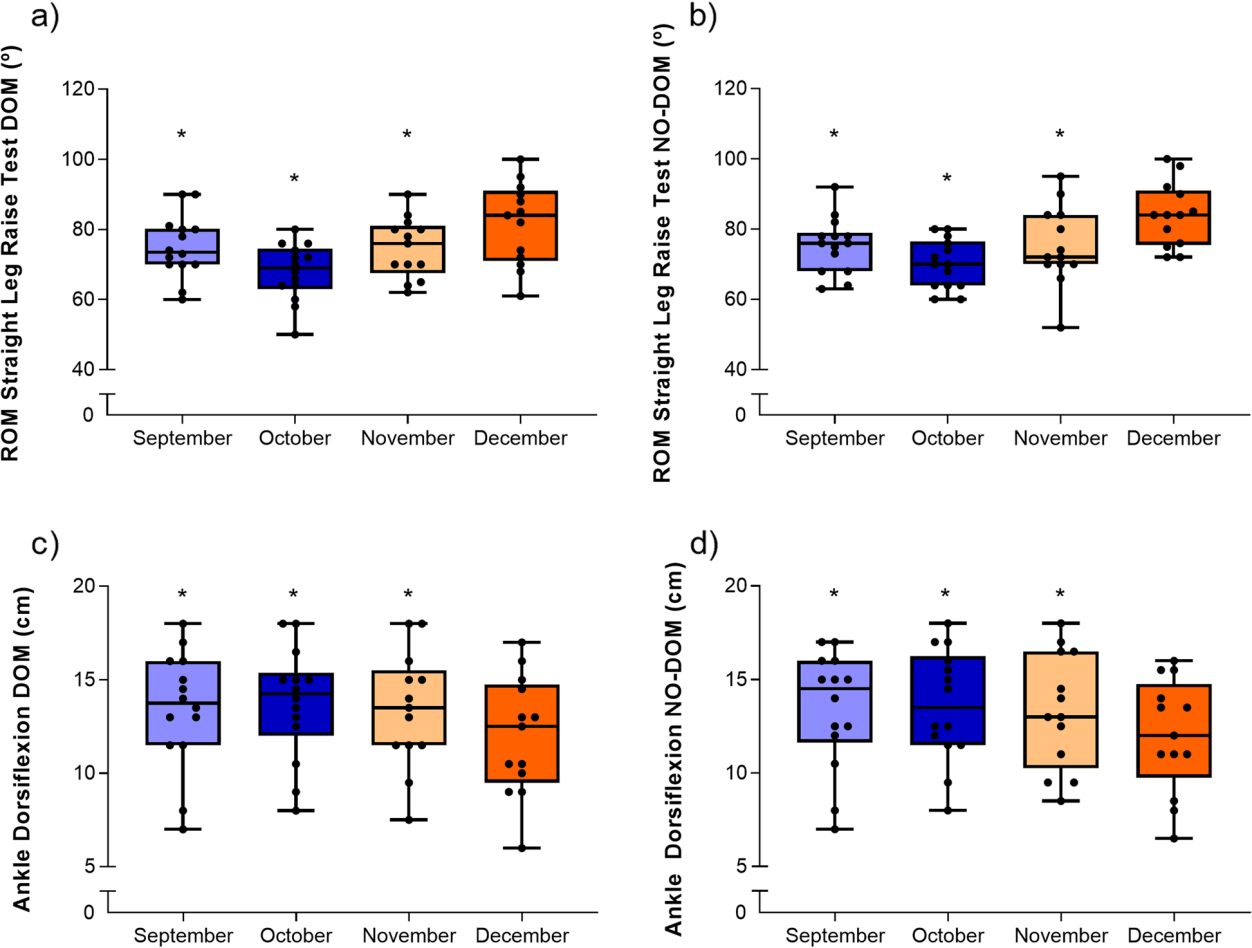
ROM values in female field hockey players. ° = degrees; cm = centimeters; DOM = Dominant; NO-DOM = non-dominant. * Significantly different when compared to December (*p* < 0.05)

Hip flexion ROM with the knee extended showed a large effect size and significant differences for the dominant limb (F_(3,33)_ = 13.8, *p* = <.001, η_p_^2^ = 0.556). The post hoc tests showed that December values were significantly higher than in previous months (i.e., September, October and November) (p = 0.002–0.030) (Fig. 3a). Similarly, for the non-dominant limb significant differences were reported (F_(3,33)_ = 435.7, *p* = <.001, η_p_^2^ = 0.523) with post hoc tests confirming higher values at December compared to the previous months (i.e., September, October and November) (*p* = 0.001–0.004) (Fig. 3b).

Ankle dorsiflexion ROM showed significant, differences over time with lower values at December compared to September, October and November in both the dominant (F_(3,33)_ = 6.64, p = 0.001, η_p_^2^ = 0.376) (Fig. 3c) and the non-dominant limb (F_(3,33)_ = 6.65, *p* = 0.001, η_p_^2^ = 0.377) (Fig. 3d).

### Countermovement jump

Countermovement jump height also varied across the 4 months (F_(3,33)_ = 9.62, *p* = <.001, η_p_^2^ = 0.490) with significant differences between values at September, October, and December versus November (*p* = 0.005–0.010).

### Perceived well-being questionnaire

No significant differences were found in fatigue parameters (χ^2^ = 2.59, df = 3, *p* = 0.459, Kendall’s W = 0.067), sleep (χ^2^ = 3.42, df = 3, *p* = 0.331, Kendall’s W = 0.087), soreness (χ^2^ = 4.01, df = 3, *p* = 0.261, Kendall’s W = 0.103), stress (χ^2^ = 2.78, df = 3, *p* = 0.426, Kendall’s W = 0.071), mood (χ^2^ = 6.52, df = 3, *p* = 0.089, Kendall’s W = 0.167), and total perceived well-being questionnaire measurement (χ^2^ = 4.85, df = 3, *p* = 0.183, Kendall’s W = 0.124) in the 4 months measured (Table 2).

**Table 2:** Items obtained in the perceived well-being questionnaire (5-WQ) in female field hockey players

Variable (Arbitrary Units)	September	October	November	December	*p-value*	Kendall’s W
Fatigue	3.43 ± 0.85	3.59 ± 0.53	3.51 ± 0.55	3.70 ± 0.89	0.459	0.066
Sleep	3.61 ± 0.59	3.71 ± 0.68	3.39 ± 0.33	3.58 ± 0.70	0.331	0.087
Soreness	3.32 ± 0.80	3.54 ± 0.67	3.23 ± 0.55	3.26 ± 0.72	0.261	0.103
Stress	3.46 ± 1.26	3.10 ± 0.94	3.08 ± 0.64	3.41 ± 1.24	0.426	0.071
Mood	3.32 ± 1.10	3.67 ± 0.58	3.59 ± 0.40	4.00 ± 0.74	0.089	0.167
Total Wellness	3.43 ± 0.54	3.52 ± 0.48	3.36 ± 0.35	3.59 ± 0.66	0.183	0.124

## Discussion

The aim of this study was to examine the variation in a range of indicators, proposed to contribute to modifiable intrinsic injury risk, during 4 months of the competitive season in semi-professional female field hockey players. These variables include objective physical measures; hamstring and adductor and abductor isometric muscle strength, hamstring and ankle dorsiflexion ROM and neuromuscular performance (i.e., measured by a countermovement jump) as well as subjective perceptual fatigue (measured by a perceived well-being questionnaire).

The main motivation reason for conducting this investigation was based on previous studies that suggested that both acute [12] and congestion related [10] demands associated with field-hockey matches provoked changes in isometric muscle strength and ROM values. However, it remains unknown to what extent these risk variables are modulated during a chronic period such as several months during the field-hockey competitive season. The main findings of this study were isometric muscle strength, ROM values and neuromuscular performance generally increased progressively during the season while no significant changes were reported in fatigue perceived levels (i.e., measured by perceived well-being questionnaire) and finally a reduction in dorsiflexion ROM values was reported.

The present data revealed that isometric knee flexion torque values increased after 4-months of a competitive season compared to the pre-season values (i.e., September) in the dominant and non-dominant limbs (39.0–43.1%) respectively. This outcome agrees with previous studies that reported an increment (12.3%) of knee isometric hamstring muscle strength in semi-professional football players after 18 weeks of the competition season (comparing pre-season vs mid-season) [31]. A possible explanation for the observed increment in isometric knee flexion torque during the 4-month follow-up could be attributed to the players starting the preseason in September after 3-months of a transition period characterized by complete cessation of all training activities which could influence a decrement in physiological and biomechanical adaptations [32]. Referring to the isometric hip values our data agrees with a previous study developed by Wollin et al. (2018) that stablished that hip isometric strength was lowest at pre-season testing and gradually increased as players adapted to the new and higher training demands [19]. However, the findings regarding hip abductor and adductor strength are contradictory to a previous study in semi-professional male football players reported changes in isometric abductor strength between pre to mid-season (July vs January) while no differences were reported in isometric adductor strength [33]. This differences between studies could be attributed to the different team-sports analyzed (i.e., field hockey vs football) and methods used.

An adequate ROM in the lower limbs is essential in field hockey due to the continuous flexed positions and multidirectional movements that field hockey players carry out during training and competitions [34, 35]. Although previous studies have analyzed the acute [12] or congestion [10] effects on ROM values in female hockey players, to the best of our knowledge, no previous investigation has evaluated the longitudinal changes in hamstring and ankle flexibility during several months of the competitive season in female field hockey players. Our data showed statistical increasements in hip flexion ROM levels with the knee extended for the dominant and non-dominant limb after 4-months (December) compared with previous months (September,October and November) reporting differences with previous studies that no reported differences across the season in semi-professional football players [31], the differences reported between studies may be partially explained by the different sports modalities compared. However, the difference obtained between studies could be explained by the sample characteristics (male vs female) or physical demands between sports (football vs field hockey). Regarding ankle dorsiflexion ROM values, a statistical decrement was observed comparing December to previous months which is in agreement with previous studies in semi-professional football players, where a decrement of 8.9–9.6% was reported for the dominant and non-dominant limbs respectively [27]. A possible reason for these decrements in ankle dorsiflexion ROM could be attributed to chronic muscle adaptations related to the demands associated with continuous training/matches during the competitive season in conjunction with the high number of accelerations/ decelerations realized as well as changes-of-direction and jumping that involve high-intensity eccentric actions increasing muscle/tendons stiffness [36] and decreasing joint ROM [37].

Strength and muscle power have been associated with success in field hockey performance due to the potential for increments in strength and power to produce faster ball speeds and shoot more powerfully [38, 39]. Thus, monitoring changes in strength/muscle power along the season is crucial for controlling the neuromuscular performance in players [40] and for detecting their fatigue status [41]. Although multiple devices (e.g. isokinetic machines, linear position transducers) have been proposed to evaluate these neuromuscular changes, in the last years the use of the countermovement jump has become popular due to the low cost, simplicity, and ease of implementation. Our data findings showed that countermovement jump levels were lower in November compared to previous (i.e., September and October) and following months (i.e., December) which could be linked to a higher number of field hockey games played during the November comparing the other months. These results contradict previous studies in team sports disciplines (e.g. football) that did not show changes in jump capacity during the in-season period [42]. These can be explained by the differences in the monitoring period (1-week period in Malone et al. vs 4-months here) and the differences between participants (young male vs female).

Perceived well-being questionnaire has been proposed as a non-invasive method for assessing players’ perceptual responses during training or competition events [13]. According to the perceived well-being questionnaire, no significant changes were found in the present study during the 4-months (16-weeks) periods in the measured fatigue variables (e.g. sleep, soreness, stress, and mood). There were only small differences observed (effect size < 0.3). These results are in agreement with a previous study that reported no evidence of long-term reductions over the seven-week mesocycle [43] in team sport athletes. Thus, perceived well-being questionnaire could be less sensitive than a countermovement jump for monitoring changes in female field hockey during a 4-month period.

Aside from its strengths, which include the prospective design, the rigorous assessment protocol and good data completeness, the current investigation has several limitations that should be discussed to enhance its applicability to real sports context scenarios. Firstly, the initial investigation had to be cut short because of the COVID-19 pandemic which led to a complete cessation of activities for over 6 months. As a result, the changes obtained in the short-time intervention period (16 weeks) could be different compared to the possible changes provoked during an entire competitive season of 9 months. Secondly, since only a single cohort of a limited number of female field hockey players participated in the study, the findings may not be readily extended to males or youth players.

## Conclusion

Increments in isometric muscle strength and fluctuations in ROM values and vertical jumping capacity are reported over an in-season mesocycle (i.e., 4 months of the competitive season). Specifically, isometric adductors, abductors and hamstrings strength progressively increase in female field hockey players during a fourth months mesocycle while ankle dorsiflexion ROM decreases. In addition, neuromuscular performance levels determined through the countermovement jump test analysis, varied with a greater load exhibited when more matches were disputed. This information is useful as strength coaches and physiotherapists can target recovery strategies to make them more efficient. Thus, the regular monitoring of modifiable intrinsic risk factors can be useful to provide information on the status of the female field hockey players.

## Data Availability

Full data for this research is available through the corresponding author upon request.
